# A clinical scoring system to predict long-term arthralgia in Chikungunya disease: A cohort study

**DOI:** 10.1371/journal.pntd.0008467

**Published:** 2020-07-21

**Authors:** Laise de Moraes, Thiago Cerqueira-Silva, Victor Nobrega, Kevan Akrami, Luciane Amorim Santos, Cibele Orge, Paula Casais, Lais Cambui, Rita de Cássia Pontello Rampazzo, Karen Soares Trinta, Camila Amato Montalbano, Maria Jania Teixeira, Luciano Pamplona Cavalcante, Bruno B. Andrade, Rivaldo Venâncio da Cunha, Marco Aurélio Krieger, Manoel Barral-Netto, Aldina Barral, Ricardo Khouri, Viviane Sampaio Boaventura

**Affiliations:** 1 Instituto Gonçalo Moniz (IGM)—Fundação Oswaldo Cruz (Fiocruz) Bahia; 2 Faculdade de Medicina da Bahia—Universidade Federal da Bahia, Salvador-BA, Brazil; 3 University of California, San Diego, Division of Infectious Disease, Department of Medi- cine, San Diego, California, United States of America; 4 Instituto de Biologia Molecular do Paraná, Curitiba, PR, Brasil; 5 Fundação Oswaldo Cruz, Bio-Manguinhos, Rio de Janeiro, RJ, Brazil; 6 Faculdade de Medicina, Universidade do Mato Grosso do Sul, Campo Grande- MS, Brazil; 7 Faculdade de Medicina, Universidade Federal do Ceará, Fortaleza-CE, Brazil; 8 Fiocruz, Campo Grande, MS, Brazil; 9 Instituto Carlos Chagas—ICC/Fiocruz, Curitiba-PR, Brazil; 10 Instituto Nacional de Ciência e Tecnologia de Investigação em Imunologia, São Paulo- SP, Brazil; 11 Rega Institute for Medical Research, KU Leuven, Leuven, Belgium; 12 Serviço de Otorrinolaringologia do Hospital Santa Izabel/Santa Casa de Misericórdia da Bahia (HIS/SCMBa), Salvador, Brazil; CDC, UNITED STATES

## Abstract

**Background:**

Chikungunya virus (CHIKV) has caused worldwide epidemics that impose a major burden on health systems. Approximately half of infected individuals develop chronic debilitating arthralgia, affecting their quality of life. Here, we identified the relevant clinical and demographic variables in the acute phase of CHIKV infection prospectively linked to chronic arthralgia to elaborate a prognostic scoring system.

**Methods:**

Acute CHIKV infection cases (n = 134) confirmed by serology or molecular test were examined <10 days of disease onset and followed for one year to evaluate for disease progression. Potential risk factors for chronic arthralgia were evaluated by multivariate analysis to develop a prognostic scoring system, which was subsequently tested in an independent validation cohort consisting of 42 individuals.

**Results:**

A total of 107 out of 134 (80%) acute CHIKV-confirmed cases from the derivation cohort were re-examined one year after enrollment. Chronic arthralgia post-CHIKV infection was diagnosed in 64 (60%). Five of the 12 parameters evaluated in the acute phase were statistically associated with persistent arthralgia and were further tested by Bayesian analysis. These variables were weighted to yield a prognosis score denominated SHERA (Sex, Hypertension, Edema, Retroocular pain, Age), which exhibited 81.3% accuracy in predicting long-term arthralgia post-CHIKV infection in the derivation cohort, and 76.5% accuracy in the validation cohort.

**Conclusions:**

The simplified and externally validated prognostic scoring system, SHERA, is a useful method to screen acutely CHIKV-infected patients at elevated risk of chronic arthralgia who will benefit from specific interventions. This tool could guide public health policies, particularly in resource-constrained settings.

## Author summary

Debilitating articular pain frequently occurs as a consequence of Chikungunya virus infection, affecting individuals' quality of life for a long time. Here we present a simple tool to predict people at risk to remain with long-term articular pain after Chikungunya infection. We evaluated 134 patients acutely affected by Chikungunya virus searching for characteristics previously described as related to persistent symptoms. We examined about 80% of those individuals after one year to identify those with persistent arthralgia. We detected five features that represent good predictors and developed a scoring system named SHERA (www.sheracalculator.com/shera), the acronyms of **S**ex (female), **H**ypertension, **E**dema, **R**etroocular pain and **A**ge (>26y). SHERA can correctly predict 8 out of every 10 Chikungunya infected individuals that will persist with articular pain symptoms at least one year after disease onset. These results were confirmed in the second group of patients from another city affected by Chikungunya outbreak.

The easy-to-use scoring system can be applied in areas with limited access to health support, helping to identify the ones who will need special care and benefit from early intervention.

## Introduction

Debilitating manifestations are attributed to infection by the arthritogenic chikungunya alphavirus (CHIKV). Until early 2000, sporadic outbreaks of chikungunya had been reported in Africa and South Asia.[1] After 2004, the virus spread to Europe and the Americas, highlighting potential for worldwide CHIKV dissemination.[2,3] In Brazil, several outbreaks have been reported since the first case in 2014, with progressively higher numbers of affected individuals with every successive epidemic.[4]

The acute phase of CHIKV infection, reported to be symptomatic in approximately 90% of infected individuals, is characterized by rapid onset of fever, with intense and polyarticular arthralgia, edema, cutaneous rash, pruritus, headache, nausea, retroocular pain and oral lesions.[5] Notably, up to 87.2% of affected patients have persistently recurrent chronic skeletal muscle symptoms and arthralgia lasting more than three months.[6–8] Chronic manifestations post-CHIKV infection can lead to absenteeism with significant social and economic impacts.[9,10]

As no vaccine or effective treatment is available, chronic symptoms persisting after the acute phase of CHIKV infection require special care.[11] Musculoskeletal deformities have been described, in addition to aggravation of comorbidities, such as hypertension and diabetes.[11–13] Renal failure and gastric complications related to the prolonged use of medications to treat pain, as well as depression, have been reported.[11,14,15]

Early identification of individuals at risk for chronic manifestations subsequent to CHIKV infection may optimize medical care. Prior work suggests that symptoms present during the acute phase relate to chronicity and that persistent arthralgia occurs more frequently in women, individuals over 40 years of age and those with preexisting comorbidities, such as hypertension.[7,16–18] However, no attempt has been made to develop a clinical/sociodemographic scoring system to predict chronicity following acute CHIKV infection.

Herein, we performed periodic evaluations of clinical symptoms up to one year in two cohorts of patients presenting clinically-, molecular- and/or serologically-confirmed CHIKV mono-infection in the acute-phase. We then sought to determine predictors of long-term arthralgia post-CHIKV infection to derive an easy-to-use prognostic scoring system.

## Materials & methods

### Ethics statement

This study was approved by the Institutional Review Board of the School of Medicine—Federal University of Bahia—Brazil (derivation cohort—approval number: 1.657.324) and by the Institutional Review Board of Bahia State University of Feira de Santana (validation cohort–- approval number: 1.450.762).

### Study population

A total of 230 individuals were recruited by active case detection in the derivation cohort between 2016 and 2018 in the context of three concomitant outbreaks of arbovirus fever (Zika/dengue/chikungunya). Two sites were selected in the state of Bahia (North: Campo Formoso and South: Itabuna) and one in the state of Ceará (Maranguape) (S1 Fig). The two furthest sites were over 1,000 km distant from each other (S1 Fig).

Our validation cohort was composed of 42 cases who met eligibility criteria from 219 CHIKV cases in Feira de Santana (Bahia State), site of the first outbreak of CHIKV in Brazil in 2014.[19] Patients were recruited at a health care unit and followed for twelve months.

Individuals were considered eligible if they reported feeling feverish, demonstrated fever or presented cutaneous rash in association with at least one of the following manifestations: cutaneous pruritus, arthritis, arthralgia, headache, myalgia or retroocular pain (ROP). In both cohorts, individuals were excluded if: 1. disease onset was longer than 10 days prior to time of recruitment; 2. no sample was collected; 3.they tested negative for CHIKV by qRT-PCR and ELISA; 4. another concomitant arbovirus infection was detected by qRT-PCR or serology in blood, saliva or urine sample.

To identify possible early clinical markers of disease progression, all participants were questioned about the presence and duration of fever, maculopapular cutaneous rash (MPCR), arthralgia, articular edema in the upper and lower extremities, ROP, myalgia, tendon pain and other comorbidities. All patients underwent a clinical examination by one or two physicians to assess acute signs of CHIKV infection such as rash and articular edema in addition to prior comorbidities, such as hypertension, previous arthropathy and chronic peripheral edema. To determine the evolution of arthralgia, these questions and clinical examination were repeated at three and 12 months after onset of clinical symptoms in individuals with confirmed CHIKV mono-infection. Individuals with confirmed CHIKV infection were classified as chronic arthralgia post-CHIKV infection if they reported continuous or weekly episodes of new musculoskeletal pain or aggravation of prior arthritic pain (in the case of reported comorbidity). All those with chronic arthralgia had one of the following, by history and physical exam: persistent arthritis, tenosynovitis, and edema. Symptomatic treatment of acute and chronic symptoms was performed, focused on pain relief as previously described.[11]

### Quantitative Reverse Transcription PCR– qRT-PCR

EDTA-treated plasma, urine and oral swab samples were collected for molecular diagnosis of ZIKV, DENV and CHIKV infection. RNA was extracted from plasma (140μL), urine (140μL) and saliva (oral swab was incubated in 140μL of H2O nuclease free) samples using a commercial Viral RNA isolation kit (QIAmp Viral RNA mini kit, Qiagen- Germany). Quantitative RT-PCR was performed using two different assays: IBMP KIT BIOMOL ZDC (IBMP-Brazil) or CDC Trioplex assay (CDC, USA) for ZIKV, CHIKV, DENV and RNAse P. All assays were performed in accordance with manufacturer's instructions.

### Serological testing

Heparin-treated plasma samples were collected for serological diagnosis of ZIKV, DENV and CHIKV infection. ELISA assays (Euroimmun, Germany) for detection of IgM antibodies against ZIKV, DENV and CHIKV were performed according to manufacturer's instructions.

A confirmed case was defined as a positive test for one of the laboratory criteria: a positive qRT-PCR in serum, saliva or urine, or detection of specific IgM antibody.

### Statistical analysis

Kolmogorov-Smirnov normality test determined the choice of parametric or non-parametric testing using the Chi-squared test, Fisher’s exact test, One-way ANOVA, the Kruskal–Wallis test. Receiver-operator (ROC) curve analysis was used to discretize continuous variables. To evaluate evolution of arthralgia, Kaplan-Meier survival curves and log-rank testing were employed. All statistical tests were two-tailed with a significance level of 5%.

All variables significant by univariate analysis were applied in compatible classifier algorithms using the following linear and non-linear approaches: Neural Network, C5.0 algorithms, Classification and Regression Tree (CART), Bayesian Network, Logistic Regression, Quick Unbiased Efficient Statistical Tree (QUEST) and Chi-squared Automatic Interaction Detection (CHAID). Each variable’s coefficient by multivariate analysis was used to build a predictive clinical scoring model. The performance parameters were calculated using the results predicted by clinical scoring system following formulas: $Sensitivity=\frac{True\;Positive(TP)}{TP+False\;Negative(FN)};\;Specificity=\frac{True\;Negative(TN)}{False\;Positive(FP)+TN};$ $Positive\;Predictive\;Value:\frac{TP}{TP+FP};\;Negative\;Predictive\;Value=\frac{TN}{TN+FN};\;Accuracy=\frac{TP+TN}{TP+TN+FP+FN}$. The confidence intervals for sensitivity, specificity, predictive values, and accuracy were calculated using the Clopper-Pearson method.

Data were analyzed using IBM SPSS version 25 (Armonk, NY: IBM Corp.), IBM SPSS Modeler 18.2 (Armonk, NY: IBM Corp.) and GraphPad Prism 7.0 software (GraphPad Software, USA).

## Results

### Description of derivation cohort

**Fig 1:**
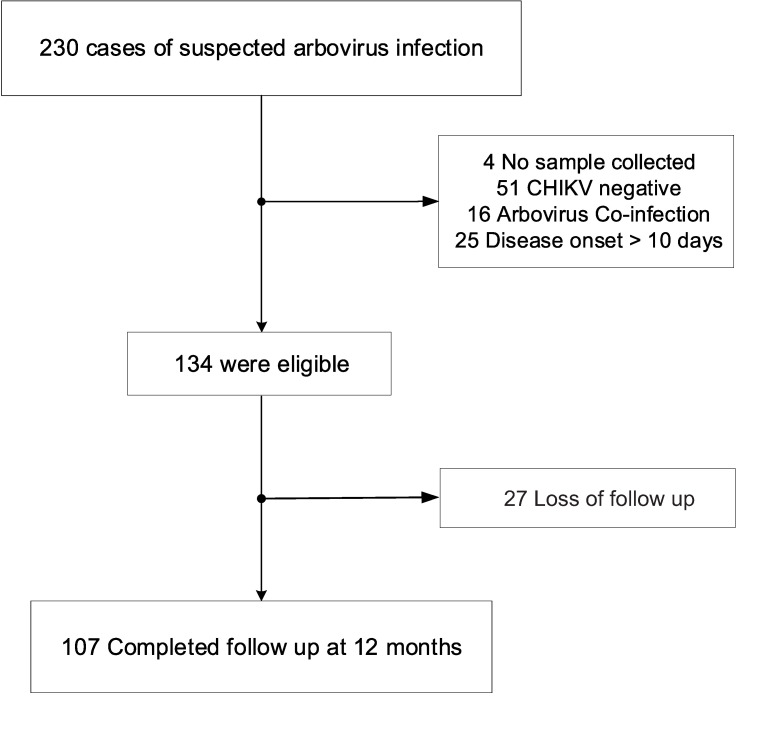
Flow chart of derivation cohort detailing exclusion criteria and number of cases excluded at each step. Follow-up conducted at 12 months after onset of CHIKV symptoms.

**Fig 2:**
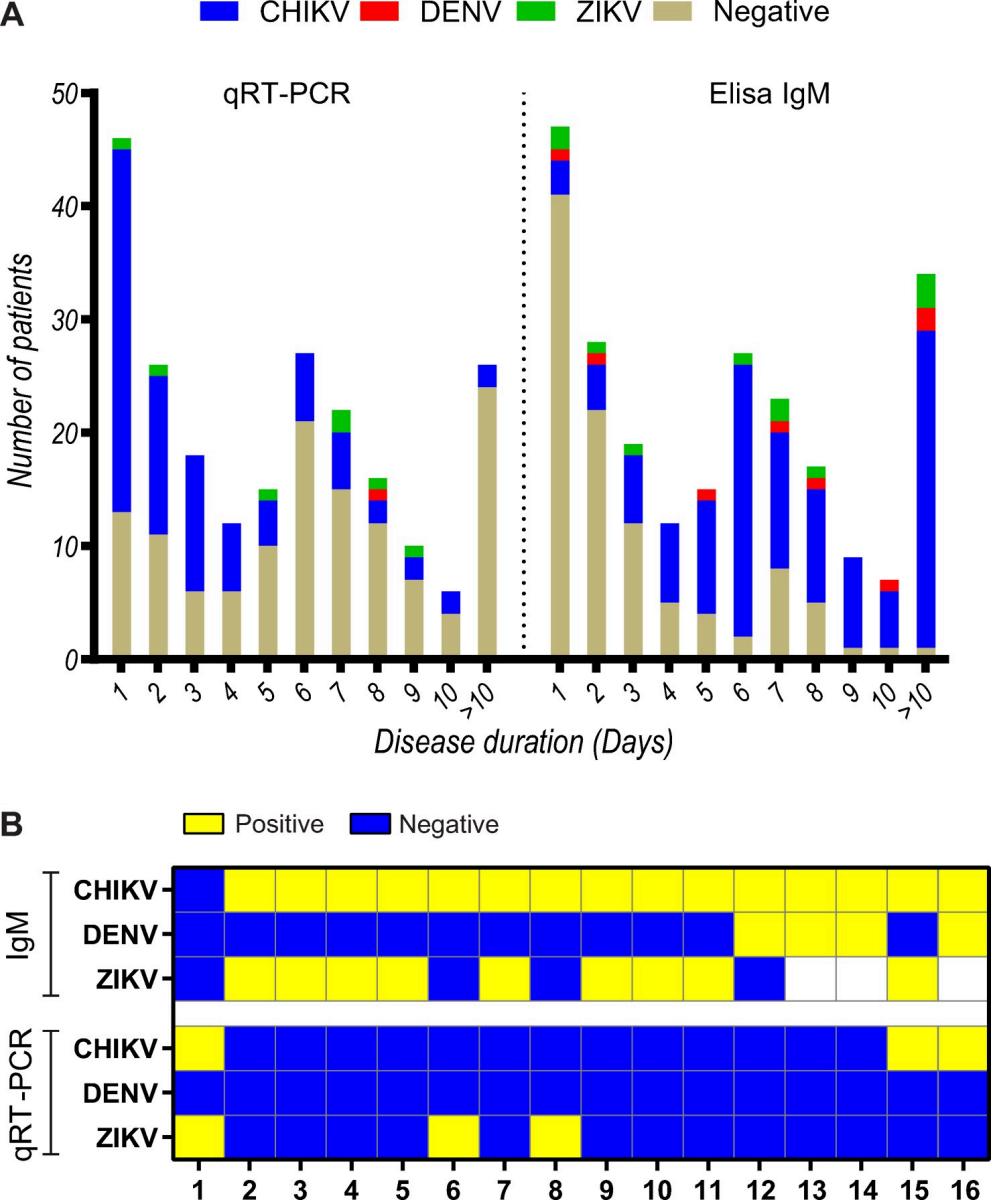
(A)Diagnostic results of arbovirus infection (chikungunya, dengue and Zika virus) performed in the study population (all 230 suspected cases). Molecular diagnosis was performed by qRT-PCR in EDTA-plasma, urine and saliva samples and serological diagnosis was based of detection of IgM by ELISA in Heparin-plasma samples. Positive CHIKV cases were represented in blue, DENV in red and ZIKV in green. Cases tested negative for all three arbovirus were represented in brown. (B) Heat map showing diagnostic results of the patients excluded due to arbovirus co-infection. Positive test (yellow), negative (blue) and not tested (white).

Among the 230 individuals initially screened, 96 patients were excluded for reasons listed in Fig 1, yielding 134 exclusively infected with acute CHIKV (Fig 1). Of these, 60.1% of CHIKV cases tested positive by qRT-PCR, 57.1% by serology and 17.3% by both methods (Fig 2A). A total of 16 cases were excluded due to ZIKV (n = 11) and DENV (n = 5) co-infection (Fig 2B). Most prevalent symptoms included arthralgia in all patients, followed by fever, headache, myalgia, edema, vomiting, ROP, MPCR, pruritus and hypertension. Of note, similar demographic data and clinical symptoms were observed at all recruitment sites (Table 1). A total of 107 patients were reevaluated after 12 months as 27 patients (20%) were unavailable for follow up. Persistent arthralgia was detected in 64 (60%) individuals (Table 1).

**Table 1:** Baseline characteristics of patients comprising derivation cohort according to study site.

Observations	Campo Formoso (n = 76)	Itabuna (n = 24)	Maranguape (n = 34)	*χ*^2^ or Kruskal Wallis	Total (n = 134)
**State**	Bahia	Bahia	Ceará		
**Year of recruitment**	2016	2016	2017		
**Disease duration (days)**	5 (2–7)	2 (1–3)	5 (2–8)	0.001	4 (2–6)
**Patient characteristic**					
Age (years)	42.0 (28–54.0)*	33.0 (23.0–43.0)^+^	43.5 (25.3–63.3)	0.419	39.0 (26.5–55)^□^
Sex (Female)	64.5% (49)	41.7% (10)	61.8% (21)	0.134	59.7% (80)
Fever	96.1% (73)	100% (24)	94.1% (32)	0.331	96.3% (129)
Myalgia	89.5% (68)	83.3% (20)	85.3% (29)	0.679	87.3% (117)
Arthralgia	100% (75)	100% (24)	100% (34)	1	100% (133)
Edema	69.7% (53)	54.2% (13)	76.5% (26)	0.188	68.7% (92)
MPCR	53.9% (41)	50% (12)	73.5% (25)	0.105	58.2% (78)
Pruritus	53.9% (41)	41.7% (10)	55.9% (19)	0.510	52.2% (70)
Headache	89.5% (68)	95.8% (23)	79.4% (27)	0.139	88.0% (118)
ROP	65.8% (50)	70.8% (17)	50.00% (17)	0.189	62.7% (84)
Vomits/Nausea	66.7% (50)*	62.5% (15)	76.5% (26)	0.469	68.4% (91)
Hypertension	21.1%(16) ^&^	8.3%(2)	20.6%(7)	0.302	18.7% (25) ^□^
**Arthralgia persistence at 12 months**	70.0%(42/60)	38.9%(7/19)	57.1%(16/28)	0.112	60.7% (65/107)

*n = 75

^+^n = 23.

^□^n = 132

^&^ n = 74

MPCR = Maculopapular Cutaneous Rash

ROP = Retro-orbital pain

### Clinical symptoms associated with persistent arthralgia after 12 months of follow-up

**Fig 3:**
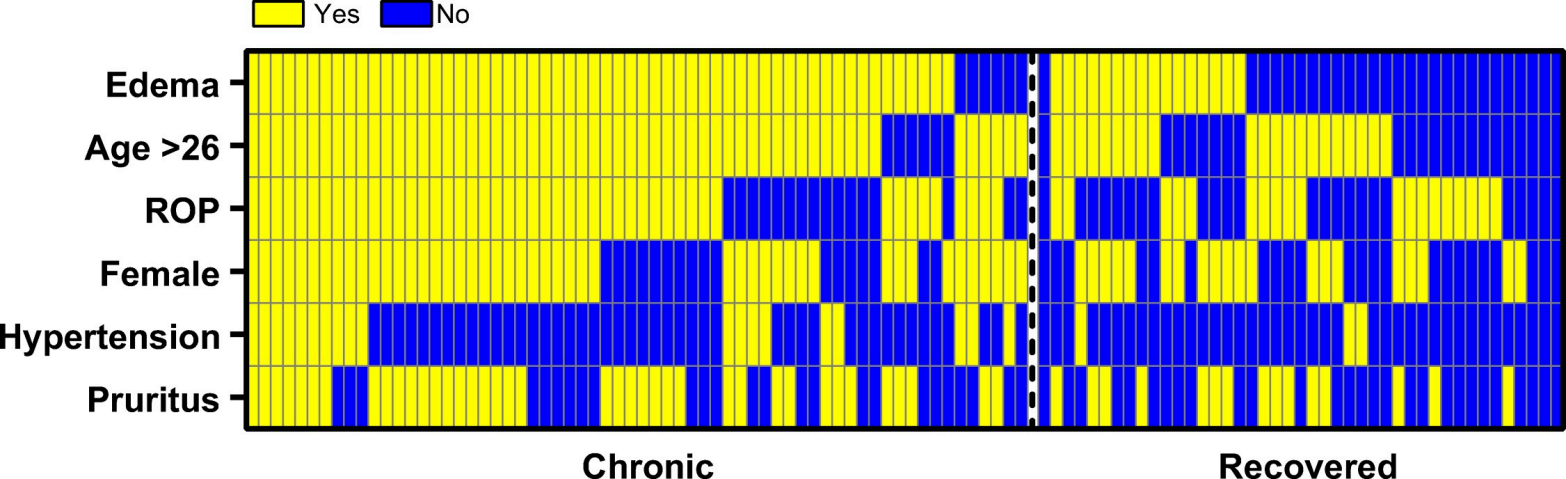
Heat map showing the presence (yellow) or absence (blue) of clinical and social demographic characteristics in individuals with chronic (n = 65) or recovered (n = 42) arthralgia. Data were collected in the first 10 days of disease onset.

Of 12 demographic and clinical parameters investigated, edema (<0.0001), pruritus (p = 0.0178), hypertension (p = 0.0065), ROP (p = 0.0036), sex (p = 0.0014) and age (dichotomized at 26 years, p<0.0001) were statistically associated with persistent arthralgia at 12 months (Table 2). Age was dichotomized using area under curve (AUC) = 0.692 and Youden Index = 0.392 (S2A and S2B Fig). Among these statistically significant observations, edema demonstrated the highest odds ratio (OR = 13.46; CI = 4.827–32.91, p<0.0001), highlighting its importance in development of chronic arthralgia after confirmed acute CHIKV infection (Table 2). A heat map graph was generated to summarize the impact of age, hypertension and other clinical signs and symptoms found to be significantly associated with chronic arthralgia (Fig 3).

**Table 2:** Performance measures of significant variables in the derivation cohort.

Observations	Sensitivity (CI)	Specificity (CI)	Positive Predictive Value (CI)	Negative Predictive Value (CI)	Likelihood Ratio	Odd’s Ratio	Fisher's exact test
Edema	89.23% (79.40% to 94.68%)	61.90% (46.81% to 75.00%)	78.38% (67.73% to 86.23%)	78.79% (62.25% to 89.32%)	2.34	13.46 (4.827 to 32.91)	<0.0001
Age >26 years old	89.23% (79.40% to 94.68%)	50.00% (33.36% to 62.28%)	73.42% (62.76% to 81.91%)	75.00% (56.64% to 87.32%)	1.79	8.286 (2.990 to 20.44)	<0.0001
ROP	73.85% (62.05% to 82.98%)	54.76% (39.95% to 68.78%)	71.64% (59.91% to 81.03%)	57.50% (42.40% to 71.49%)	1.63	3.418 (1.461 to 7.844)	0.0041
Sex (Female)	72.31% (60.42% to 81.71%)	52.38% (37.72% to 66.64%)	70.15% (58.34% to 79.77%)	55.00% (39.83% to 69.29%)	1.54	2.872 (1.239 to 6.427)	0.0139
Hypertension	29.23% (19.58% to 41.20%)	92.86% (80.99% to 97.54%)	86.36% (66.67% to 95.25%)	45.88% (35.70% to 56.42%)	4.09	5.370 (1.510 to 17.97)	0.0065
Pruritus	60.00% (47.86% to 71.03%)	64.29% (49.17% to 77.01%)	72.22% (59.11% to 82.38%)	50.94% (37.88% to 63.88%)	1.68	2.700 (1.205 to 6.001)	0.0178

ROP = Retro-Orbital Pain

**Fig 4:**
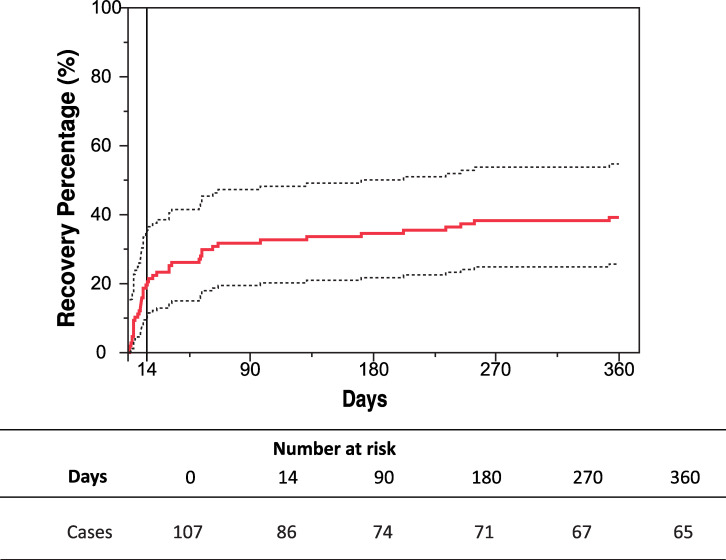
Recovery percentage of CHIKV-infected patients over the time. Recovery percentage was analyzed by Kaplan-Meier survival curve (solid line) and 95% confidence intervals (dashed line). The numbers at risk represent the number of cases with arthralgia at each time point.

A Kaplan-Meier analysis of frequency of recovery reveals that from 42 patients that were recovered at day 360, 21 (50%) were asymptomatic at day 14 (Fig 4).

### Clinical predictors of persistent arthralgia after 12 months of follow-up

To better understand the independent values of each variable in classification of possible cases of chronic arthralgia, multivariate analysis was performed in the derivation cohort, considering all variables with *p*<0.05 (Fisher’s exact test) and multinomial logistic models were built adding variables sequentially. The quality of each model was assessed by Akaike information criterion (AIC). Accordingly, pruritus was excluded as addition of this variable in the model worsened its performance.

The remaining variables were subsequently used in all compatible classifier algorithms. The top three models based on AUC and accuracy were the Bayesian Network, Logistic Regression and Classification and Regression Tree (CART). All three models showed similar results (S3 Fig). The Bayesian Network model presented the best overall accuracy (83.02%, AUC = 0.921; S3 Fig).

**Fig 5:**
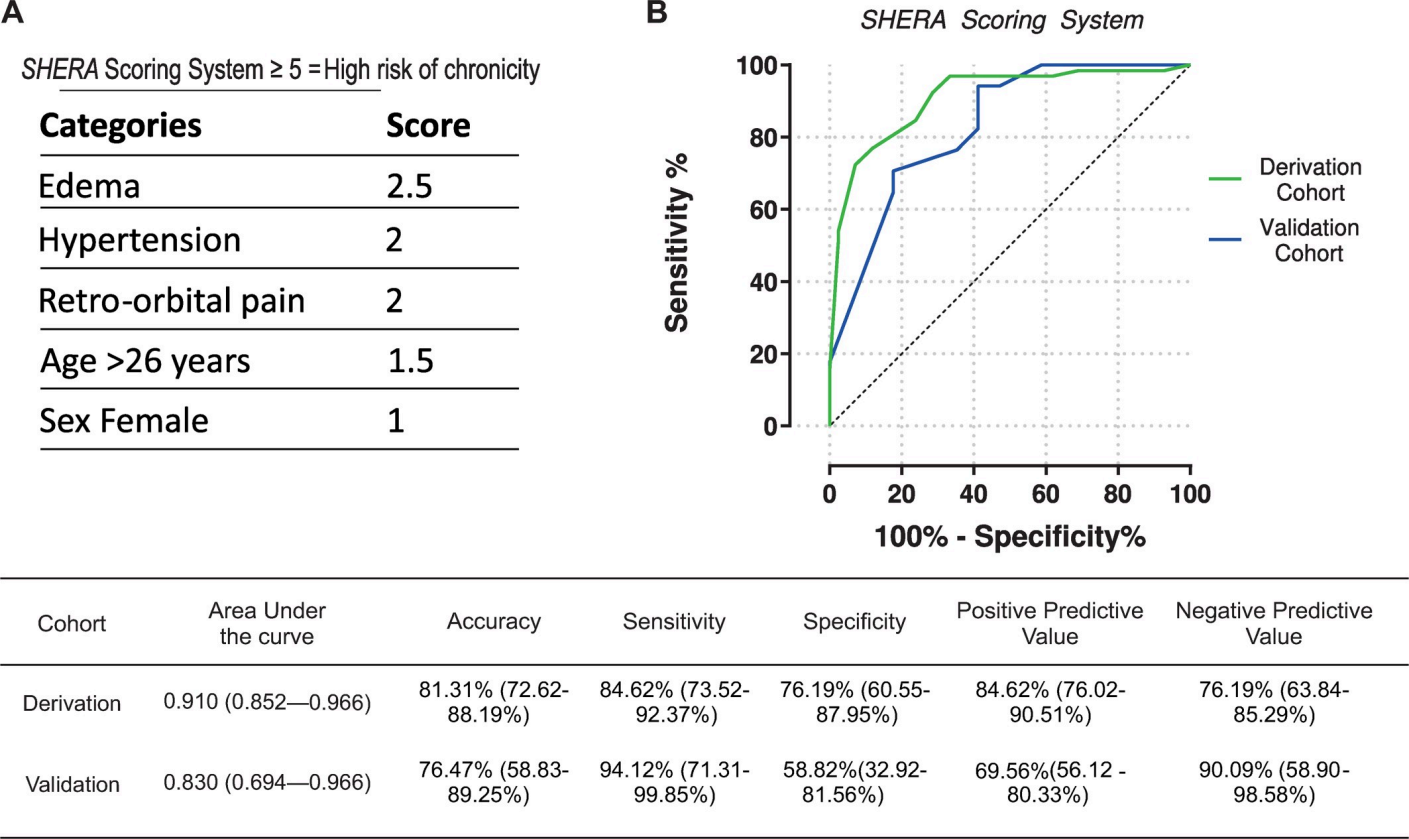
(A) SHERA Scoring System categories and score. (B) Performance measures of SHERA Scoring System (with the 95% confidence interval values in parentheses) in derivation and validation cohorts.

The variable coefficients calculated by the Bayesian Network model were multiplied by 10 and used to build the predictive clinical scoring model. The nearest integer value was used to assign a weight to each independent predictor. The resulting values were Edema = 2.5 points, ROP = 2, Hypertension = 2, Age>26 = 1.5, Female = 1 (Fig 5A). This five-factor prognostic clinical scoring scale, named SHERA score (**S**ex, **H**ypertension, **E**dema, **R**etroocular pain, **A**ge), presented an AUC of 0.909 (95%CI 0.852–0.966) and was split into two risk categories (i.e., low and high risk of chronicity) according to the best accuracy indicated by Youden Index. The best cutoff point was found to be ≥ 5 points, indicating a high risk of chronicity, while a score < 5 indicated low risk. We also built an online tool to calculate the SHERA score value at www.sheracalculator.com/shera. The resulting performance measures in the derivation cohort were 81.31% (95%CI 72.62–88.19) accuracy, 84.62% (73.52–92.37) sensitivity, 76.19% (60.55–87.95) specificity, 84.62% positive predictive value and 76.19% negative predictive value (Fig 5B).

### Validation Cohort

The same diagnostic criteria used in the derivation cohort were applied to select 42 patients included in the validation cohort. Baseline characteristics were similar to the derivation cohort, as demonstrated in Table 3. Within the first 10 days of disease onset, fever, myalgia and headache were more frequently reported, followed by MPCR, edema, pruritus, ROP, vomiting and hypertension. Eight out of the 42 (19.05%) were lost to follow up, yielding 34 patients reevaluated after 12 months. Persistent arthralgia was identified in 17 patients (50.00%) (Table 3 and S4 Fig).

**Table 3:** Comparison of baseline characteristics of patients from both cohorts.

Observations	Derivation Cohort (n = 134)	Validation Cohort (n = 42)	x^2^ or Kruskal Wallis
**Year of recruitment**	2016–2017	2015	
**Disease duration (days)**	4 (2–6)	4 (2–6)	0.577
**Patient characteristics**			
Age (years)	38.0 (26–55)*	39.0 (28.5–47)	0.778
Sex (Female)	59.7% (80)	61.9% (26)	0.799
Fever	96.3% (129)	92.9% (39)	0.355
Myalgia	87.3% (117)	92.9% (39)	0.319
Arthralgia	100% (134)	100% (42)	1
Edema	68.7% (92)	81.0% (34)	0.124
MPCR	58.2% (78)	81.0% (34)	0.0075
Pruritus	52.2% (70)	61.9% (26)	0.272
Headache	88.0% (118)	81.0% (34)	0.250
ROP	62.7% (84)	64.3% (27)	0.851
Vomits/Nausea	68.4% (91)	54.8% (23)	0.107
Hypertension	18.7% (25) *	16.7%(7)	0.770
**Arthralgia persistence**	60.7% (65/107)^&^	50.0%(17/34) ^+^	0.272

*n = 132

MPCR = Maculopapular cutaneous rash

ROP = Retro-Orbital Pain

The application of the SHERA scoring model to predict persistence of arthralgia in the validation cohort resulted in an overall accuracy of 76.47% (58.83–89.25%) ([0.830 AUC (0.694–0.966)], with 94.12(71.31–99.85)% sensitivity, 58.82(32.92–81.56)% specificity, 69.57% positive predictive value and 90.91% negative predictive value (Fig 5B).

## Discussion

The present study derived a simplified clinical scoring system shown to accurately predict chronic arthralgia post-CHIKV infection in a Brazilian cohort of 107 individuals, and further validated externally in an independent cohort of 42 patients. Only individuals with CHIKV mono-infection were included in the study as other arbovirus infections were excluded by antibody and molecular testing of serum, saliva and urine samples. Furthermore, only acute cases with initial evaluation conducted within 10 days of disease onset were included to minimize recall bias.

The components of the SHERA scoring system have not been previously combined to compose a prognosis system. It is important to note that all clinical and demographic markers identified in the derivation cohort, used to compose the SHERA scoring system, had been previously linked to persistent symptoms as a consequence of CHIKV infection.[7,16,20–24] Age was the most frequently mentioned predictor of persistent arthralgia, with a range of 20–60 years presenting increased risk of developing chronic arthralgia post-CHIKV infection.[7,16,17,21,22] This reported variability in age range may be related to demographic differences among patients recruited, as well as study design. Increased risk in older populations has been reported in studies based on telephone or e-mail interviews, excluding individuals younger than 18 years.[7,17] This strategy may have missed younger patients, thereby increasing the median age of the observed population. In our cohorts, active recruitment and clinical evaluation by a physician at all time-points ensured accurate representation of all age groups. Almost 25% of the patients recruited herein were under 26 years of age, and only 25% presented with symptoms one year after disease onset, compared to 73.42% of individuals older than 26 years. Similarly, a prior study demonstrated chronicity in 17% of individuals aged < 25 years and increased risk of chronicity in those over 25 years of age.[24]

A higher proportion of women (72,31%) was found in the group with chronic arthralgia post-CHIKV infection compared to those who recovered after acute disease (47,62%). Concordantly, previous studies have reported an increased risk of long-term arthralgia in women. [20,22,25]

Edema, hypertension and ROP were also related to the persistence of chronic arthralgia post-CHIKV infection. Development of edema at early stages of infection may be a hallmark of an intense anti-CHIKV immune response or proliferative viral activity in the joints that ultimately results in chronic tissue damage and arthritis.[26] However, prior arthropathy or peripheral vascular disease should be investigated to exclude any other possible causes of edema, especially in the lower extremities. Furthermore, hypertensive individuals were also more likely to persist with arthralgia, as previously reported.^7^ This condition also seems to be associated with more severe outcomes and increased mortality in the acute phase of CHIKV infection.[27,28] Together, these data suggest the possible synergistic action of an arthritogenic virus with other inflammatory conditions that compound clinical manifestations. While we speculate that both edema and ROP in individuals who develop persistent arthralgia post-CHIKV infection may be indicative of exacerbated systemic inflammation during the acute phase, comprehensive investigation of such a mechanism has yet to be performed.

Among the group of patients who recovered and did not develop chronic arthralgia post-CHIKV infection, approximately 60% reported being asymptomatic within a month of initial symptoms. This may be linked to rapid viral clearance due to effective early mechanisms of immune defense that seem to influence clinical recovery.[29]

While our study has multiple strengths, a potential limitation may be that both derivation and validation cohorts are from the same region and with relatively small sample size. While they were recruited during outbreaks from different years, with a rigorous inclusion criteria, it is possible that there is genetic variability of patients and differences in CHIKV genotypes that could affect the accuracy of the proposed score. Although the variables used as part of the parsimonious score have been individually published worldwide previously, the robustness of the SHERA score in patients requires additional evaluation with a larger sample size in distinct geographic areas at risk for CHIKV infection. Additionally, specificity seemed to be the weakest parameter of SHERA Scoring System specially in the validation cohort, suggesting this prognosis tool may be used primarily for screening and it may be improved by adding novel biomarkers.

While no specific treatment is available for CHIKV, the identification of a precise tool that predicts those at risk for chronicity could significantly impact patient care. Individuals at risk for chronicity would benefit from a multidisciplinary therapeutic approach and careful monitoring of comorbidities potentially aggravated by CHIKV infection. Given that CHIKV has an elevated attack rate and high predilection for chronicity, the SHERA score could aid in predicting the frequency and distribution of chronic cases, effectively targeting public health services in at risk areas and mitigating the socioeconomic impact of arthralgia post-CHIKV infection.

This study highlights the reliability of a novel validated easy-to-use clinical score for risk of chronicity in CHIKV infection. As a simple and inexpensive tool that uses clinical information, this prognostic scoring system may be effective in limited resource settings and aid in guiding public health policies to alleviate the social economic impacts of CHIKV infection.

## Supporting information

S1 Fig Study areas for the derivation (Campo Formoso, Itabuna and Maranguape) and validation (Feira de Santana) cohorts.
(TIF)

S2 Fig A) Performance of age to predict arthralgia after one year using Area Under the Curve analysis. B) Relative values of chronic arthralgia with respect to age groups.
(TIF)

S3 Fig Performance of multivariate models designed to predict chronic arthralgia post-chikungunya infection in the derivation (A) and in the validation (B) cohorts.
(TIF)

S4 Fig Flow chart of validation cohort showing exclusion criteria and number of cases excluded at each step. Follow-up conducted 12 months after onset of CHIKV symptoms.
(TIF)
