# Supplementary figures and images for: A clinical scoring system to predict long-term arthralgia in Chikungunya disease: A cohort study
Source: PLoS Negl Trop Dis. 2020 Jul 21;14(7):e0008467. doi: 10.1371/journal.pntd.0008467 (PMC7373495; doi:10.1371/journal.pntd.0008467)

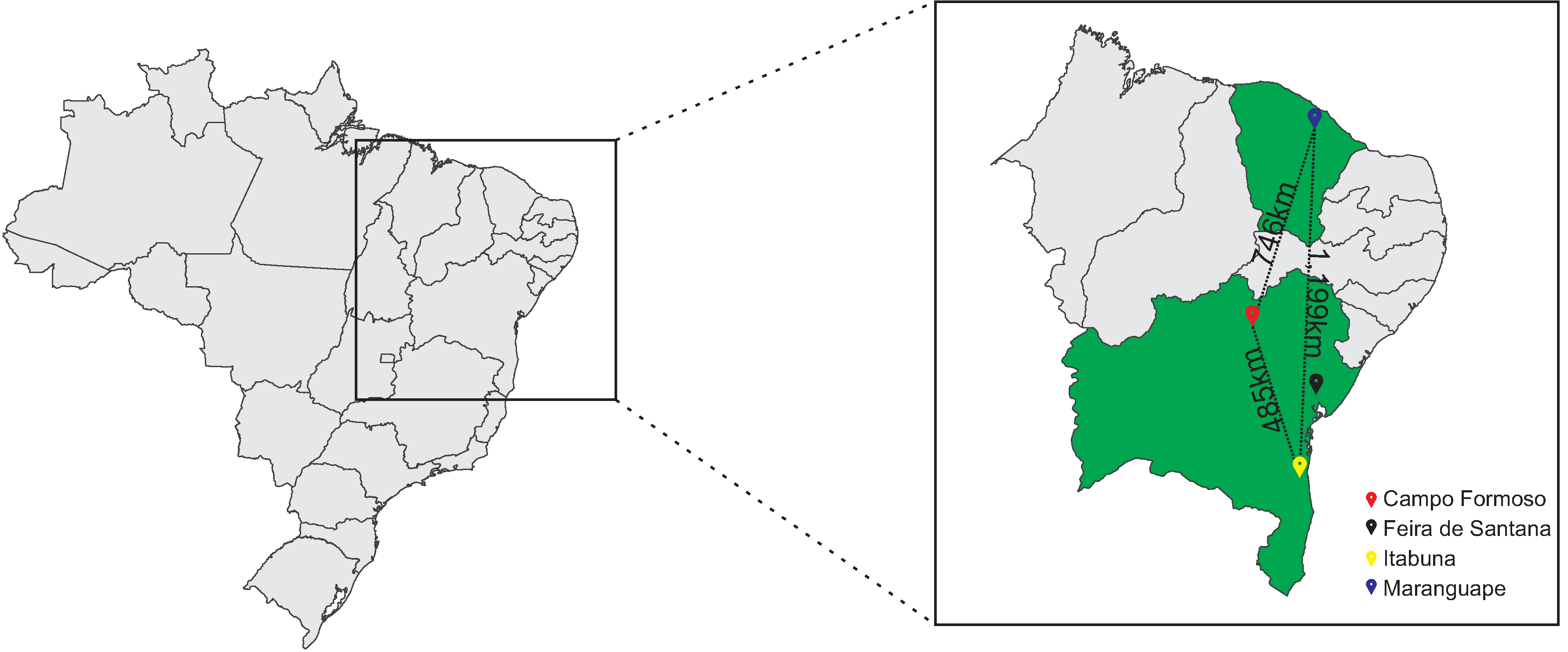

Supplement: S1 Fig — Study areas for the derivation (Campo Formoso, Itabuna and Maranguape) and validation (Feira de Santana) cohorts. (TIF) [file pntd.0008467.s001.tif]

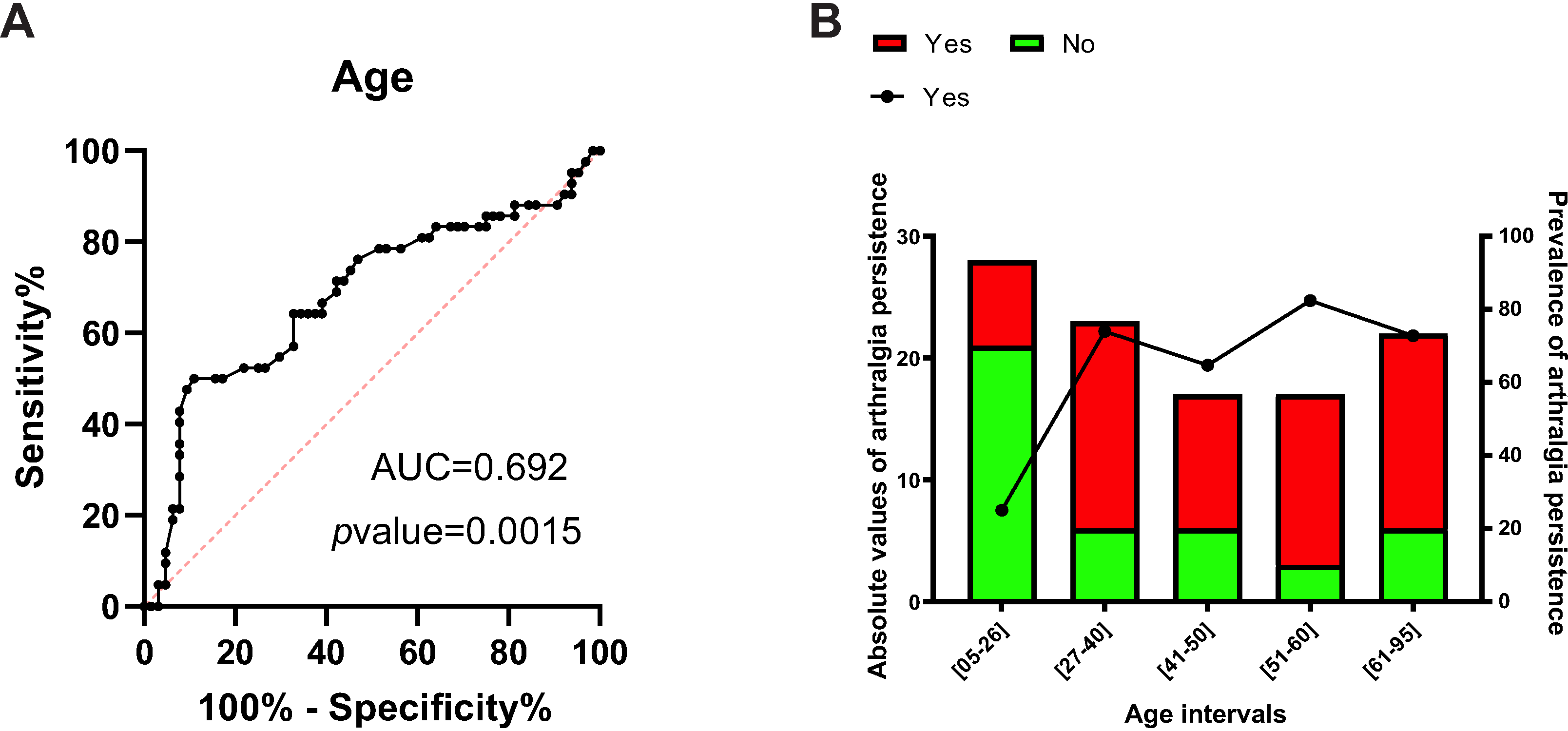

Supplement: S2 Fig — A) Performance of age to predict arthralgia after one year using Area Under the Curve analysis. B) Relative values of chronic arthralgia with respect to age groups. (TIF) [file pntd.0008467.s002.tif]

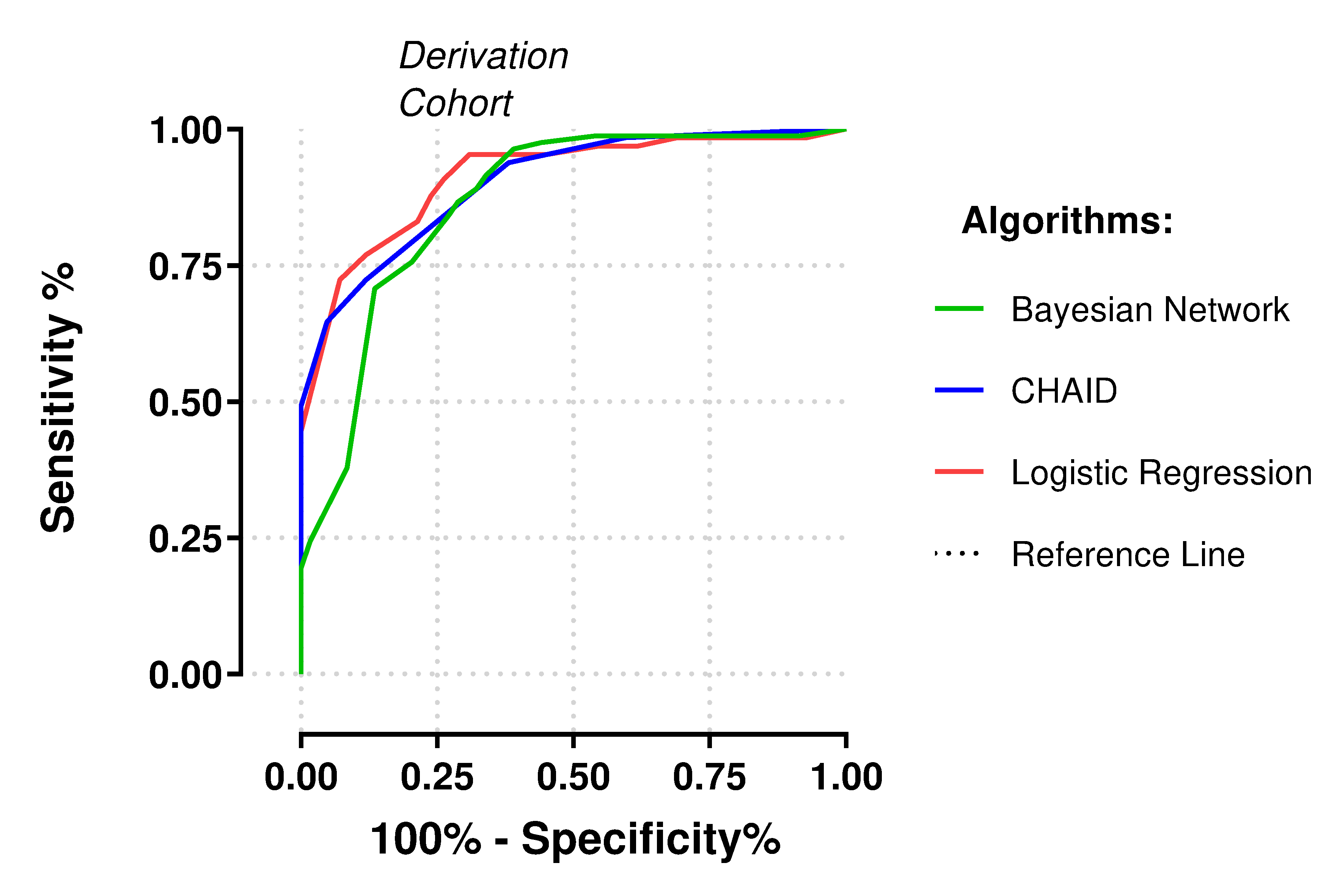

Supplement: S3 Fig — Performance of multivariate models designed to predict chronic arthralgia post-chikungunya infection in the derivation (A) and in the validation (B) cohorts. (TIF) [file pntd.0008467.s003.tif]

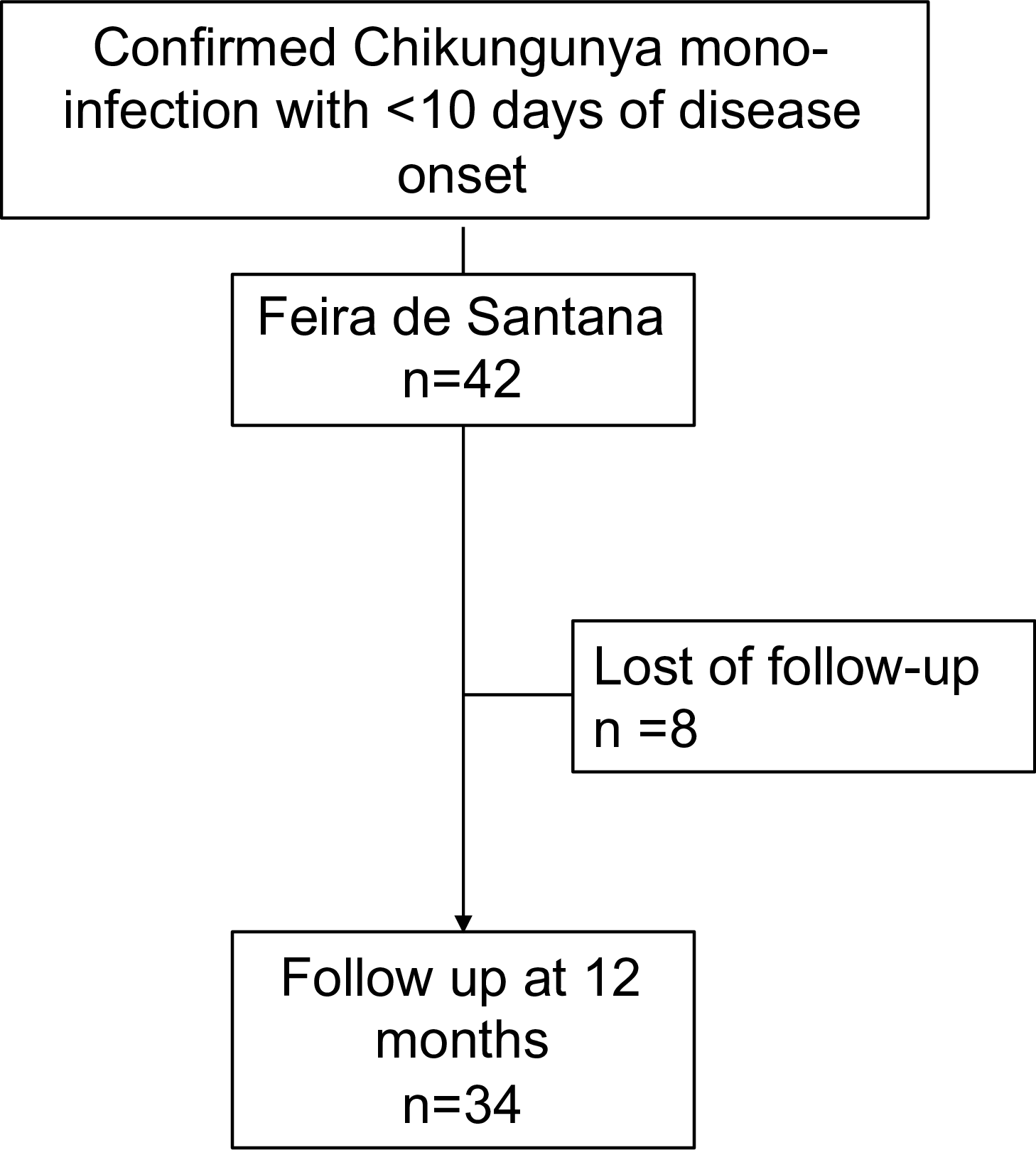

Supplement: S4 Fig — Flow chart of validation cohort showing exclusion criteria and number of cases excluded at each step. Follow-up conducted 12 months after onset of CHIKV symptoms. (TIF) [file pntd.0008467.s004.tif]
